# *Stenotrophomonas maltophilia*: the need for randomized controlled trials to evaluate treatment options

**DOI:** 10.1128/asmcr.00127-25

**Published:** 2025-09-11

**Authors:** Ya-Ting Chang, Luca Mezzadri, David L. Paterson

**Affiliations:** 1 ADVANCE-ID, Saw Swee Hock School of Public Health, National University of Singapore 37580, Singapore, Singapore; 2 Division of Infectious Diseases, Department of Internal Medicine, Kaohsiung Medical University 38023, Kaohsiung, Taiwan; 3 School of Medicine and Surgery, University of Milano-Bicocca 9305, Milan, Italy; 4 Infectious Diseases Translational Research Programme, Yong Loo Lin School of Medicine, National University of Singapore 37580, Singapore, Singapore; Pattern Bioscience, Austin, Texas, USA

## Abstract

The most optimal treatment for serious *Stenotrophomonas maltophilia* infections remains undetermined based on current evidence. The evolution of Infectious Diseases Society of America (IDSA) guidance from 2022 to 2024 reflects growing support for combination therapy and a shift away from agents with unfavorable pharmacokinetic/pharmacodynamic profiles, such as tigecycline. No randomized controlled trials (RCTs) have been conducted to date, and existing observational comparative studies have yielded heterogeneous results. Despite efforts to adjust for bias and confounders, definitive conclusions remain elusive, largely due to methodological limitations inherent in retrospective designs, such as immortal time bias. For serious *S. maltophilia* infections, a multi-arm trial is likely necessary to generate robust evidence; however, challenges, such as large sample size requirements, difficult patient recruitment, and high implementation costs remain significant barriers. To overcome these obstacles, innovative trial designs and analytic approaches have emerged. Target trial emulation, for instance, seeks to approximate RCT-level evidence using observational data. Several other designs aim to improve the flexibility and efficiency of clinical trials, including: multi-arm, multi-stage (MAMS) trial designs; embedding a multi-arm trial within a larger platform trial; an analysis method known as PRACTical method composed of personalized randomization treatment options relevant to individuals; and Bayesian analytic frameworks, enabling simultaneous comparison across multiple treatment arms. As we seek answers to complex clinical dilemmas, such as the life-threatening *S. maltophilia* infection reported by Qadabashi and colleagues in a recent article in *ASM Case Reports* (ASM Case Rep 1:e00005-25, 2025, https://doi.org/10.1128/asmcr.00005-25), these forward-looking trial designs and analytic methodologies will be critical in shaping the future of evidence-based treatment strategies.

## COMMENTARY

*Stenotrophomonas maltophilia* is a non-fermentative Gram-negative bacillus, which is notable for its intrinsic resistance to carbapenems. In a recent article published in *ASM Case Reports*, Qadabashi and colleagues report a case of *S. maltophilia* bio-prosthetic valve endocarditis successfully treated with mitral valve replacement surgery and the combination of levofloxacin and trimethoprim-sulfamethoxazole (1). While the need for source control (that is, removal and replacement of the infected bio-prosthetic valve) is not for debate, there is considerable uncertainty about the most appropriate antibiotic(s) for serious *S. maltophilia* infections.

This uncertainty is reflected in the evolution of Infectious Diseases Society of America (IDSA) guidance from 2022 to 2024 (Table 1). Trimethoprim-sulfamethoxazole (TMP-SMX) monotherapy is indicated in the 2022 guidance as an acceptable initial option, yet in subsequent years, only combination therapy approaches are advocated (2). TMP-SMX as monotherapy is also advocated as a treatment of choice by 2023 Spanish Society of Infectious Diseases and Clinical Microbiology (SEIMC) Guidelines (3). Although not present in IDSA guidance, other experts have advocated levofloxacin as an appropriate initial choice (with some caveats) (4). Tigecycline was present as an option (as part of combination therapy) in the 2022 and 2023 guidance but has been omitted as an option in 2024. Extrapolating from the 2024 IDSA guidance, seven possible regimens could be used for treatment of *S. maltophilia* (TMP-SMX plus levofloxacin, TMP-SMX plus minocycline, TMP-SMX plus cefiderocol, levofloxacin plus minocycline, levofloxacin plus cefiderocol, minocycline plus cefiderocol, and ceftazidime-avibactam plus aztreonam) (5). Now that aztreonam-avibactam has been registered in the United States and Europe, with other jurisdictions to follow, presumably another option is now possible (Table 2).

**TABLE 1:** Evolution of IDSA guidance for treatment of moderate to severe *S. maltophilia* infections

Year of IDSA guidance	Key points adressed in the recommendations
IDSA 2022 Guidance Any of 3 approaches were suggested:	The use of combination therapy, with TMP-SMX and minocycline as the preferred combination; The initiation of TMP-SMX monotherapy with the addition of a second agent (minocycline [preferred], tigecycline, levofloxacin, or cefiderocol) if there is a delay in clinical improvement with TMP-SMX alone; or The combination of ceftazidime-avibactam and aztreonam, when intolerance or inactivity of other agents is anticipated.
IDSA 2023 Guidance Either of 2 approaches was suggested:	The use of two of the following agents: TMP-SMX, minocycline/tigecycline, cefiderocol, or levofloxacin; or The combination of ceftazidime-avibactam and aztreonam, when significant clinical instability is evident or intolerance to or inactivity of other agents is identified.
IDSA 2024 Guidance Either of 2 approaches was suggested:	The use of two of the following agents: cefiderocol, minocycline, TMP-SMX, or levofloxacin; or The combination of ceftazidime-avibactam and aztreonam.

**TABLE 2:** Potential treatment options for serious *S. maltophilia* infections^*b*^

Recommended regimens^*a*^	Rationale
TMP-SMX tolerable	
1.0) TMP-SMX +minocycline	TMP-SMX as the preferred backbone regimen due to its extensive clinical experience and consistent favorablle *in vitro* profile.
Intravenous minocycline unavailable	
2.1) TMP-SMX + levofloxacin 2.2) TMP-SMX + tigecycline 2.3) TMP-SMX + cefiderocol	Minocycline demonstrates strong *in vitro* activity across surveillance studies, along with more reliable PK/PD data. 2.1) Levofloxacin susceptibility rates vary among regions. The combination should be avoided if local epidemiological data indicate resistance rates >10%. 2.2) While more accessible than intravenous minocycline, tigecycline has less reliable clinical outcomes and PK/PD profiles, particularly in critically ill patients. 2.3) When available, cefiderocol is a reasonable choice over the above two options for critically unstable patients.
TMP-SMX intolerable	
1.0) Minocycline + levofloxacin 1.1) Minocycline + cefiderocol or CAZ-AVI plus ATM or ATM-AVI	1.1) Reasonable choice for critically unstable patients.
Intravenous minocycline unavailable	
2.1) Cefiderocol + levofloxacin 2.2) CAZ-AVI plus ATM or ATM-AVI 2.3) Cefiderocol + tigecycline 2.4) Levofloxacin + tigecycline	2.1) Despite limited clinical experience, cefiderocol has demonstrated very low resistance to date. 2.3-4) Combination with tigecycline is less favored, as previously noted. If none of the above combinations are feasible, consider TMP-SMX rapid desensitization if possible, provided the isolate is susceptible.

Dosage recommendations for adults	
Levofloxacin	750 mg/day
TMP-SMX	10–15 mg/kg/day (trimethoprim component), divided Q8h–Q6h 15–20 mg/kg/day if critically unstable or augmented renal clearance
Minocycline	200 mg Q12h
Tigecycline	200 mg loading, then 100 mg Q12h maintenance
Cefiderocol	2 g Q8h 2 g Q6h for augmented renal clearance
CAZ-AVI plus ATM	2.5 g Q8h plus 2 g Q8h
ATM-AVI	2.67 g loading, then 2 g Q6h maintenance

^*a*^ Recommendations are presented in order of preference; step down to the next option if the preferred regimen is unavailable or not tolerated. All antimicrobial agents are recommended in intravenous formulation.

^*b*^ TMP-SMX, trimethoprim-sulfamethoxazole; CAZ-AVI, ceftazidime-avibactam; ATM, aztreonam; ATM-AVI, aztreonam-avibactam; q8h, every 8 hours; q6h, every 6 hours; q12h, every 12 hours.

We consider that if guidance by some of the world’s experts proposes at least seven possibilities for treatment, then the evidence for any of these as superior to any other regimen is far from secure. Indeed, no randomized controlled trial (RCT) has yet been performed comparing treatment options for *S. maltophilia*. Given the clear importance of this organism in serious hospital-acquired and healthcare-associated infections, we propose that there is now an urgent need for rigorous evidence generation on this topic.

There are a number of observational studies published in which comparisons of different treatment options have been made (6–18). Unfortunately, observational studies that undertake assessments of comparative effects of treatments are beset by a large number of biases and confounders. At a very intuitive level, it could be presumed that anxious physicians may be more likely to administer to sicker patients combination therapy rather than monotherapy or newer agents in preference to older agents. Most observational studies would attempt to control for this possibility by some matching method using severity of illness scores (for example, the Pitt Bacteremia Score or APACHE scores), but these may not truly reflect the composite of factors that make up an increased likelihood of poor outcome.

Another issue with observational studies is that they may not take into account biases, such as immortal time bias. Take, for example, a comparison of combination therapy with monotherapy for *S. maltophilia*. It is possible that some patients died while on monotherapy in the early days after infection onset, while survivors lived long enough to then be given combination therapy. This would bias the outcome assessment in favor of combination therapy. A relatively new technique that seeks to overcome this bias in observational studies is target trial emulation. This technique aims to draw causal inferences from observational data by designing the study to emulate a hypothetical randomized trial. As outlined by Hernán and Robins, minimizing bias requires rigorously defining the ideal trial protocol that would answer the research question and ensuring that the corresponding elements of the observational study align with the same principles (19).

The gold-standard method of comparison of different treatment options is the RCT. That no RCT has yet been performed on treatment options for *S. maltophilia* is a reflection of the potential challenges that would be faced. As noted above, there would need to be consideration of a multi-arm trial given the many options indicated in current guidelines. This would necessitate a trial with a very large sample size. One trial design that accounts for multiple treatment options is the multi-arm, multi-stage (MAMS) trial. This design has been widely used in the evaluation of treatment options for prostate cancer (20) and has been recently used in the evaluation of regimens for tuberculosis (21). Another option is embedding a multi-arm trial on *S. maltophilia* options within a larger platform trial. The TREAT-GNB platform trial has recently commenced recruiting patients. This trial evaluates antibiotic options for carbapenem-resistant Enterobacterales (CRE), carbapenem-resistant *Acinetobacter baumannii* (CRAB), and carbapenem-resistant *Pseudomonas aeruginosa* (CR-PA). It uses an analysis method known as PRACTical (22, 23). This method assumes that there is no well-defined standard of care treatment. Each enrolled patient has a “personalized randomization list” which is composed of only those treatment options relevant to that patient. For example, a patient allergic to TMP-SMX would not have TMP-SMX monotherapy or combination therapy options in their personalized randomization list. The practical method uses an analytic method analogous to network meta-analysis in comparing different treatment options.

Another possible RCT design would be to directly compare each option with each other using Bayesian analytic methods (24). Perhaps more applicable would be to compare just two, three, or four options, such as monotherapy with older agents (TMP-SMX, minocycline, and levofloxacin), monotherapy with newer agents (cefiderocol and aztreonam-avibactam), combination therapy with older agents, and combination therapy, including a newer agent.

A summary of the study designs and analytic approaches mentioned above is provided in Table 3.

**TABLE 3:** Key features and advantages of innovative clinical trial designs and analytic frameworks

Design or analytic approach	Key features	Advantages	Reference
Target Trial Emulation	Uses observational data to emulate a hypothetical randomized clinical trial, with prespecified eligibility criteria, treatment strategies, and time zero (start of follow-up).	Reduces immortal time and confounding bias. Feasible when RCTs are not viable or ethical.	(19)
Multi-Arm Multi-Stage (MAMS) Trial	Simultaneous testing of multiple treatment arms with planned interim analyzes to stop inferior options.	Adaptive and efficient. Reduces patient exposure to ineffective therapies.	(20)
Platform Trial with PRACTical Design	Perpetual trial evaluating multiple treatment options, analogous to network meta-analysis; enables personalized randomization based on clinical eligibility. Identifies and ranks a set of best available treatments.	Reflects real-world constraints. Accommodates patient heterogeneity and maximizes individual relevance. Supports indirect comparisons.	(22)
Bayesian Analytic Framework within Randomized Clinical Trials	Uses a subjective probability reflecting belief in a hypothesis, unlike frequentist methods. Generates posterior probability by integrating previous information (the prior probability) and data from the study (the observed likelihood). Enables dynamic updating of the estimated treatment effect as new data accrue and provides an intuitive representation of uncertainty through credible intervals.	Enables incorporation of prior evidence. Supports adaptive trial decisions (e.g., early stopping). Improves precision in sample size estimation. Permits a more informative interpretation of trial results beyond statistical significance versus non-significance.	(24)

A challenge in any RCT would be whether to include both bacteremia and hospital-acquired pneumonia as inclusion criteria. Most patients with *S. maltophilia* bacteremia have monomicrobial infections, whereas a sizeable proportion of those with hospital-acquired pneumonia have polymicrobial infection. The impact of the treatment of organisms other than *S. maltophilia* on outcome would need to be carefully considered. The patient reported by Qadabashi and colleagues had a bio-prosthetic valve, which needed to be replaced. In other words, “source control” needed to be performed. Any RCT on *S. maltophilia* options would have to account for the variety of source control methods that might occur in the management of these patients, such as removal of infected central venous catheters or other prosthetic devices.

Thankfully, the patient reported by Qadabashi and colleagues had a favorable outcome. However, the mortality of *S. maltophilia* infections is unacceptably high, with crude mortality rates ranging from 21% to 69% and attributable mortality reported between 12% and 37.5% (25, 26). We believe that mortality should be able to be reduced when optimal treatment for this infection is known. The inclusion of a domain of study of *S. maltophilia* treatment options within a platform, such as the TREAT-GNB trial, may be the only way in which optimal treatment can be determined.
